# Phylogeny and History of the Lost SIV from Crab-Eating Macaques: SIVmfa

**DOI:** 10.1371/journal.pone.0159281

**Published:** 2016-07-14

**Authors:** Kevin R. McCarthy, Welkin E. Johnson, Andrea Kirmaier

**Affiliations:** 1 Program in Virology, Harvard Medical School, Boston, MA, United States of America; 2 Biology Department, Boston College, Chestnut Hill, MA, United States of America; Emory University School of Medicine, UNITED STATES

## Abstract

In the 20^th^ century, thirteen distinct human immunodeficiency viruses emerged following independent cross-species transmission events involving simian immunodeficiency viruses (SIV) from African primates. In the late 1900s, pathogenic SIV strains also emerged in the United Sates among captive Asian macaque species following their unintentional infection with SIV from African sooty mangabeys (SIVsmm). Since their discovery in the 1980s, SIVs from rhesus macaques (SIVmac) and pig-tailed macaques (SIVmne) have become invaluable models for studying HIV pathogenesis, vaccine design and the emergence of viruses. SIV isolates from captive crab-eating macaques (SIVmfa) were initially described but lost prior to any detailed molecular and genetic characterization. In order to infer the origins of the lost SIVmfa lineage, we located archived material and colony records, recovered its genomic sequence by PCR, and assessed its phylogenetic relationship to other SIV strains. We conclude that SIVmfa is the product of two cross-species transmission events. The first was the established transmission of SIVsmm to rhesus macaques, which occurred at the California National Primate Research Center in the late 1960s and the virus later emerged as SIVmac. In a second event, SIVmac was transmitted to crab-eating macaques, likely at the Laboratory for Experimental Medicine and Surgery in Primates in the early 1970s, and it was later spread to the New England Primate Research Center colony in 1973 and eventually isolated in 1986. Our analysis suggests that SIVmac had already emerged by the early 1970s and had begun to diverge into distinct lineages. Furthermore, our findings suggest that pathogenic SIV strains may have been more widely distributed than previously appreciated, raising the possibility that additional isolates may await discovery.

## Introduction

During the 20^th^ century, simian immunodeficiency viruses (SIV) from African primates were transmitted to humans on no fewer than 13 occasions [1]. One event, involving an SIV from chimpanzees, initiated the global human immunodeficiency virus type-1 (HIV-1) pandemic (group M) in the early 1900s [2–4]. Three additional HIV-1 groups are the result of independent cross-species transmission events of distinct SIVs from chimpanzees (group N) and gorillas (groups O and P) [5–7], while HIV type-2 (HIV-2) groups A-I arose from at least nine independent cross-species transmissions of SIVs from sooty mangabeys [8–10]. In each instance, the emergence of an HIV likely began in a rural African population, and archived samples from the earliest period of viral adaption to humans are unlikely to exist.

In the mid 1960s, SIV strains from captive sooty mangabeys (SIVsmm) were unintentionally transmitted to macaques housed in United States primate centers [11–15]. In the 1980s and 1990s, emerging pathogenic SIVs were isolated from four macaque species: SIVmac from rhesus macaques (*Macaca mulatta*) [16]; SIVmne from pig-tailed macaques (*Macaca nemestrina*) [17]; SIVstm from stump-tailed macaques (*Macaca arctoides*) [18, 19]; and SIVmfa from crab-eating macaques (*Macaca fascicularis*) [20]. SIVmac and SIVmne and their respective hosts have since become valued non-human models for HIV pathogenesis and vaccinology. Because these viruses emerged in captive animals from which historical samples and veterinary records were archived, the study of these viruses has been useful for elucidating the process of cross-species transmission, viral adaptation, and the emergence of pathogenic immunodeficiency viruses [21].

Of the reported SIVs from captive macaques, SIVmfa was never subject to genetic characterization. We recovered the genome of this “lost” SIV from archived samples, and used sequence and phylogenetic analyses to infer its origin and establish its place in the SIV/SIVsmm/HIV-2 phylogeny. We conclude that the emergence of SIVmfa was the product of two cross-species transmission events: the SIVsmm-to-rhesus-macaque transmission that resulted in the SIVmac lineage [8, 11–13], followed soon thereafter by the transmission of SIVmac to crab-eating macaques. The chain of transmission likely involved monkeys housed in at least three primate centers: the California National Primate Research Center (CNPRC), the defunct Laboratory for Experimental Medicine and Surgery in Primates (LEMSIP) in upstate New York, and the former New England Primate Research Center (NEPRC) in Southborough, MA. Importantly, LEMSIP ceased operations in 1998 and NEPRC in 2016, and such closures may compromise future efforts to recover samples like the ones used here to re-isolate SIVmfa. We report these findings in part to encourage similar efforts to chronicle the history of these viruses.

## Materials and Methods

### Rediscovery of SIVmfa

In 1987, PBMC from animal Mf186-76 were co-culture with H9 cells. A vial containing cell supernatant from this co-culture was obtained (a gift from R.C. Desrosiers). Viral RNA was isolated using the High Pure Viral RNA Kit (Roche USA, Indianapolis, IN). Amplicon-specific cDNAs were produced using the SuperScript^™^ III One-Step RT-PCR System with Platinum^®^ Taq High Fidelity kit (Invitrogen, Carlsbad CA) using primers listed in S1 Table; amplicons were either sequenced directly, or TA-cloned using the TOPO TA cloning kit (Invitrogen, Carlsbad, CA) and then sequenced. The sequence of the full-length SIVmfa genome was deposited in GenBank under the accession number KU500642. The SIVmfa *vif* sequence was previously reported under KF030930 [22].

### Phylogenetic analyses

**Fig 1:**
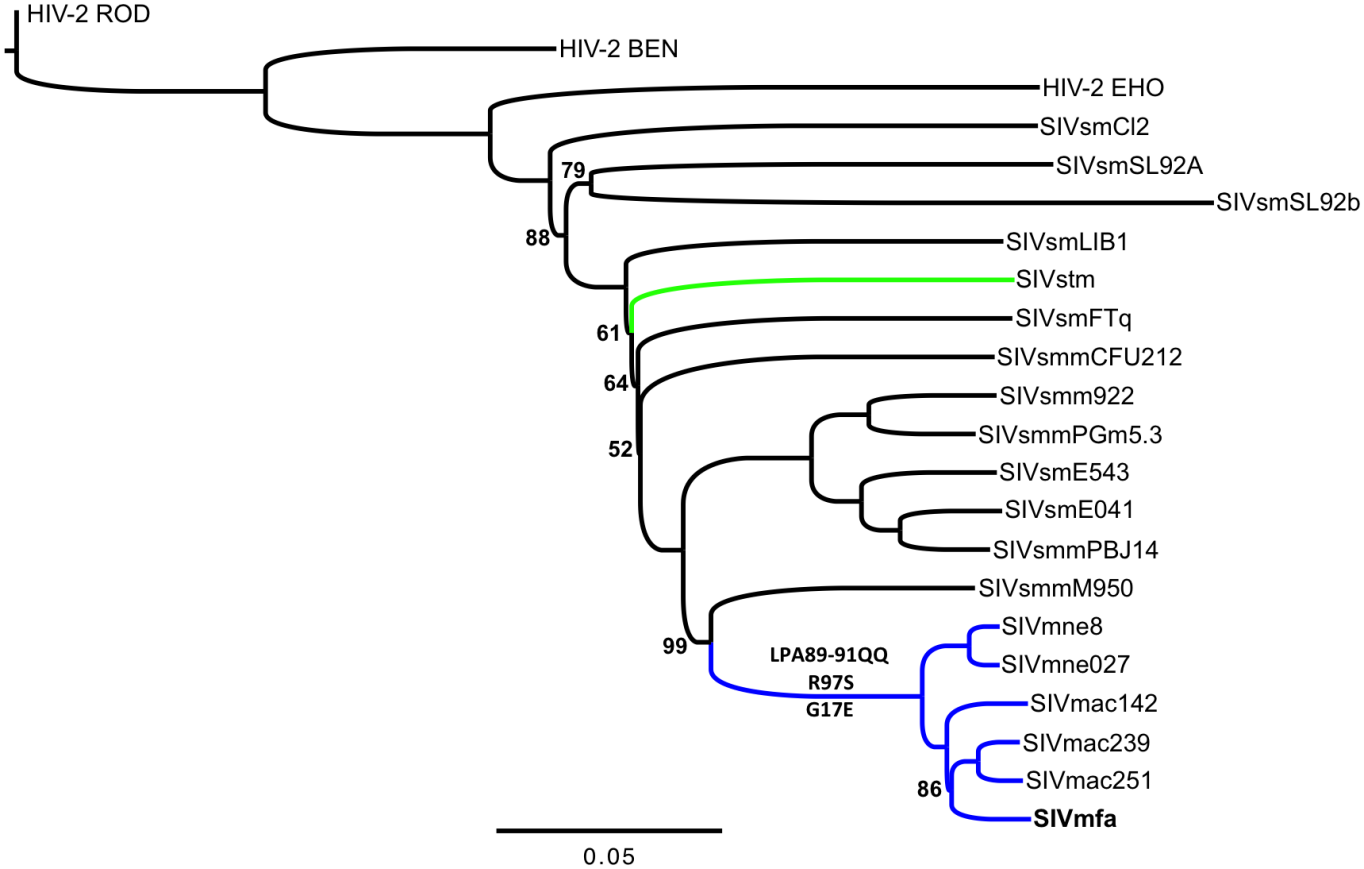
Phylogeny of SIVmfa and related SIV and HIV-2 strains. Neighbor-joining tree with 1000 bootstrap replicates of full-length proviral sequences. HIV-2 ROD was set as the outgroup, and branch support is 100 unless noted otherwise. Scale bar shows substitutions per site. The independent branching of SIVstm and all other macaque-adapted strains of SIV is highlighted in green and blue, respectively. Macaque-specific viral adaptations in CA and Vif are shown above and below the branch leading to the SIVmac/mne/mfa clade, respectively.

Viral genomic sequences (S2 Table) were aligned with the MAFFT algorithm in Geneious (version 6.1.8; Biomatters Limited, Auckland, New Zealand) and the alignments were manually inspected and adjusted where necessary. The trees are based on alignments of the following lengths: full-length provirus– 10820 bp, gag– 1707 bp, pol– 3426 bp, env– 2724 bp, vif– 657 bp, vpr– 321 bp, vpx– 343 bp, nef– 804 bp, partial gag– 265 bp, partial R3-gag– 526 bp, partial env– 482 bp. After performing a heuristic search of the alignments, parsimony trees, neighbor-joining trees and likelihood trees, rooted on HIV-2 ROD, with 1000 bootstrap replicates (parsimony and neighbor-joining) or 100 bootstrap replicates (likelihood) were generated in PAUP* (version 4.0a147) [23] (Fig 1, S1–S3 Figs, and data not shown). FigTree (version 1.4.0) was used to render trees (http://tree.bio.ed.ac.uk/software/figtree/).

## Results

### Origins and sequence of SIV from crab-eating macaques

The first known simian immunodeficiency virus, SIVmac, was isolated from rhesus macaques at NEPRC in 1985 [16]. To determine SIV prevalence among the macaque species housed at NEPRC, Daniel *et al*. screened sera from animals for reactivity with SIVmac antigens [20]. Four crab-eating macaques had sera that strongly reacted with SIVmac antigens, including Mf186-76, the only surviving animal at the time [20]. Lymphocytes were isolated from lymph node tissue of Mf186-76 and co-cultured with H9 cells, and proviral DNA from this co-culture was used to generate a lambda phage clone containing a complete provirus [20, 24]. Comparative restriction endonuclease digests indicated that the SIVmf186 clone was related to, but distinct from SIVmac clones, including SIVmac239, SIVmac251, SIVmac142, SIVmac309 and SIVmac157 [24].

The initial SIVmf186 molecular clone was never further characterized and attempts to locate it at NEPRC were unsuccessful. We obtained a single cryo-vial of supernatant of the Mf186-76 lymph-node lymphocyte/H9-cell co-culture. In an attempt to recover virus, we used half of the volume to infect HuT-78 cells. No cytopathic effects or detectable p27 were observed during six weeks of culture (data not shown), suggesting that the infectivity of the sample was severely diminished. Viral RNA was isolated directly from the remaining volume. Reverse transcribing PCR amplification of genomic segments longer than 1–2 kb was inefficient due to the degraded state of the viral RNA (likely explaining the absence of detectable infectious virus). We therefore used short amplicons overlapping by 50–150 bp to amplify and sequence the entire genome and the sequence reads, which overall exhibited a very low degree of diversity, were assembled to produce a full-length proviral sequence (see S1 Table for primer sequences). The 10,243 bp long provirus codes for the expected viral genes without any premature stop codons. Our sequence is the consensus of all overlapping PCR amplicons and we designated this isolate SIVmfa, to distinguish it from the lost SIVmfa186 clone.

We next compared the SIVmfa consensus sequence to six macaque-adapted SIV strains, twelve SIVsmm strains, and HIV-2 group A and B strains using various phylogenetic analyses (see S2 Table for accession numbers). Overall, our results agree with previously published phylogenies of the SIVsmm/SIVmac group [25], and place SIVmfa firmly in the lineage of macaque-adapted SIV strains. In both single-gene as well as whole-genome-based trees, SIVmfa is basal to the SIVmac239/SIVmac251 clade, and the combined SIVmfa/SIVmac clade, in turn, forms a sister taxon to SIVmne (Fig 1, S1 and S2 Figs, and data not shown). The nearest non-SVmac/mne relatives to SIVmfa are SIVsmmM950 and a clade consisting of SIVsmmPBJ, SIVsmE660 and SIVsmE543-3, whereas the most distantly related SIVsmm in our analysis is SIVsmSL92B with 25.6% (2,488 nt) divergence. The nearest non-SIV relatives of SIVmfa are HIV-2 groups D and H [10]. SIVstm clustering with other SIVsmm strains confirms that the transmission of SIVsmm into stump-tailed macaques was an independent cross-species transmission event [18, 19].

Two of the oldest and most SIVmac-like SIVsmm sequences ever amplified from a sooty mangebey are SIVsm77CNPRC_CFU287 and SIVsm76CNPRC_CFU287, two short fragments in the R3-*gag* and *env* regions, respectively. They were isolated from a sample archived at CNPRC in 1975–77. Phylogenetic analyses suggested that this virus is extremely closely related to the virus that founded the SIVmac/SIVmne lineage [12]. Our analysis of SIVmfa confirms that, like SIVmac and SIVmne, SIVmfa is part of that same lineage and can ultimately be traced back to cross-species transmission of SIVsmm to macaques, most likely at CNPRC (S3 Fig).

### Rhesus macaques were the reservoir for SIVmac, SIVmne and SIVmfa

While it is widely accepted that SIVsmm is the progenitor of all known macaque-adapted SIV isolates, the order of events in the chain of transmission of SIVsmm into macaques has never been fully resolved. In theory, two scenarios that are not mutually exclusive can explain the phylogenetic clustering of SIVsmm with SIVmac/SIVmne/SIVmfa: 1) animals from the three macaque species were independently infected by closely related SIVsmm strains, possibly from the same animal or animals from the same sooty mangabey colony; 2) members of one macaque species were initially infected by SIVsmm and then additional cross-species transmission events resulted in the infection of the other macaque species. In addition to our phylogenetic evidence, which favors the latter scenario, we looked for the presence of characteristic macaque-specific viral adaptations to resolve between these two possibilities. Specifically, certain alleles of the host restriction factors TRIM5 and APOBEC3G that are only found in macaques select for known viral escape mutations; these mutations thereby serve as evidence of viral evolution in a macaque host, and can be used as lineage markers.

The repertoire and functionality of restriction factors in rhesus macaques, pig-tailed macaques, and crab-eating macaques are well studied. For TRIM5, three functionally distinct allele classes have been identified: TRIM5α^Q^, TRIM5α^TFP^ and TRIM5^CypA^, which are distributed among macaques in a species-specific manner [26–31]. While TRIM5^CypA^ is found in all three macaque species, it is the only TRIM5 variant found in pig-tailed macaques [28–34] TRIM5α^Q^ and TRIM5α^TFP^ alleles are present in both rhesus and crab-eating macaques but the relative frequencies of these alleles in the two aforementioned species are dramatically different. In captive rhesus macaques, TRIM5α^TFP^ occurs at roughly 55% [35]. In contrast, in crab-eating macaques, TRIM5α^TFP^ is seen at less than 1% (no more than five occurrences in almost 600 alleles sampled, including animals from different colonies and diverse geographic origins) [30, 32, 36–43].

**Fig 2:**
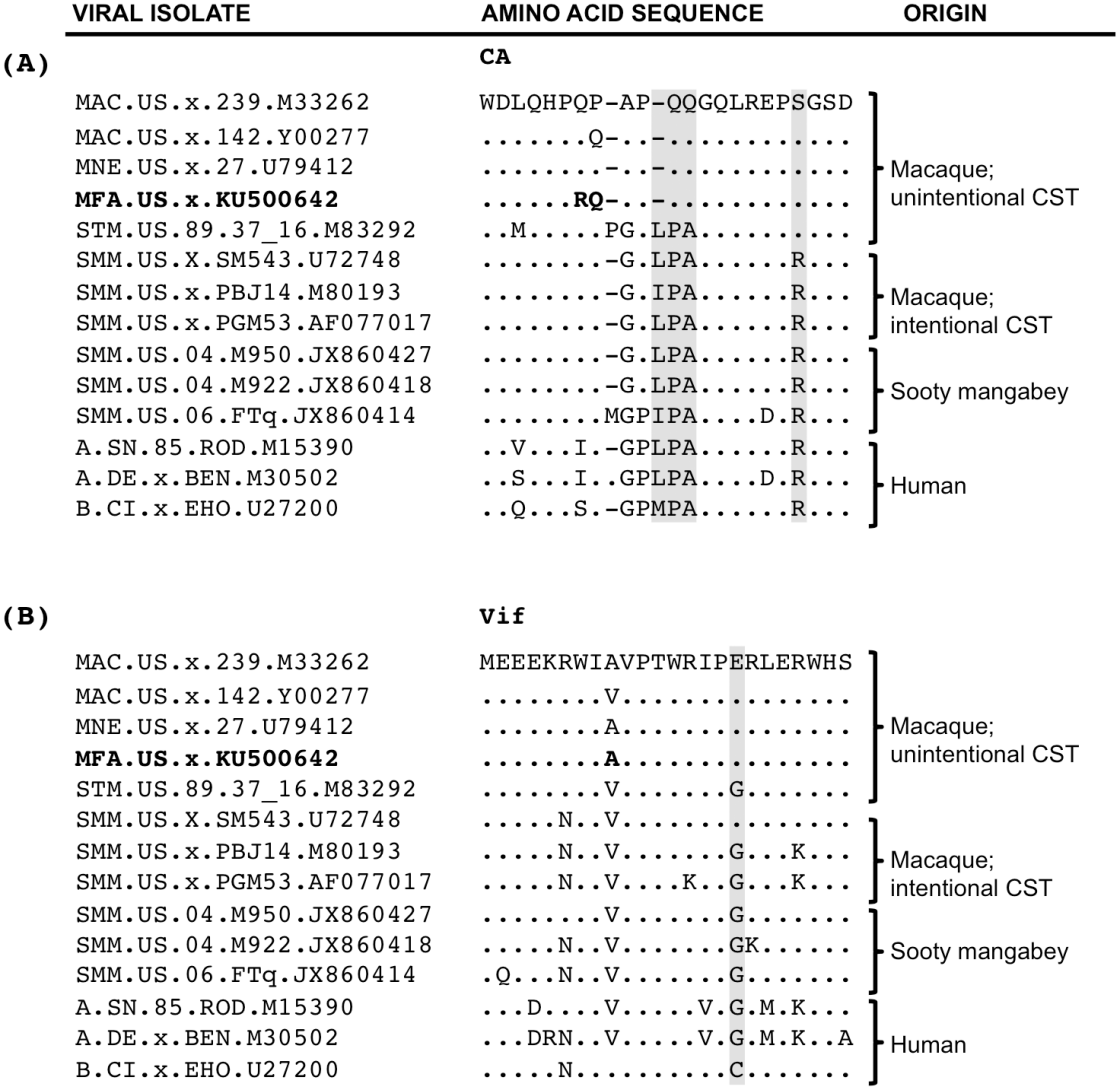
Macaque-specific adaptations in SIV CA and Vif. Comparison of SIVmfa protein sequence (bolded) to related isolates of SIV and HIV-2. (A) Residues 79–100 of CA (based on SIVmac239 numbering) representing strains used in Fig 1. Adaptation to rhesus macaque TRIM5^TFP^ (R97S) and TRIM5^CypA^ (IPA/LPA89-90QQ) are highlighted in grey. (B) Residues 1–24 of Vif representing strains used in Fig 1. The G17E adaptation to rhesus macaque APOBEC3G^LR^ is highlighted in grey.

Both TRIM5^CypA^ and TRIM5α^TFP^ can restrict SIVsmm and HIV-2 isolates [27–29, 34, 44, 45]. Accordingly, the emergence of macaque-adapted SIV required mutations to evade these TRIM5 variants [44, 46]. In SIVmac, escape from TRIM5^CypA^ occurred via alteration of an LPA/IPA at capsid (CA) position 89–91, which is conserved among SIVsmm and HIV-2 isolates [44]. In SIVmac, SIVmne and SIVmfa, LPA/IPA was replaced by QQ, which is highly conserved in this lineage (Fig 2A). We note that LPA/IPA-to-QQ is a complicated multi-nucleotide substitution/deletion event, which likely is difficult to produce for the virus, and it is therefore a strong indicator for extended positive selection pressure exerted on SIVmac, SIVmne and SIVmfa by macaque TRIM5^CypA^. The SIVmac/SIVmne/SIVmfa lineage also shares an R-to-S substitution at capsid position 97 that is involved in resistance to TRIM5α^TFP^-mediated restriction [44, 46]. CA R97 is highly conserved in SIVsmm and HIV-2 isolates, while CA S97 is highly prevalent in SIVmac/SIVmne/SIVmfa isolates, as well as in SIVstm (Fig 2A). Of note, pig-tailed macaques lack TRIM5^TFP^ and in crab-eating macaques this allele is very rare, yet both SIVmne and SIVmfa encode the S97 TRIM5 escape mutation. The simplest explanation for this is that the ancestor or ancestors of SIVmne and SIVmfa replicated in rhesus macaques, in which TRIM5^TFP^ is the most abundant TRIM5 variant.

The emergence of macaque-adapted SIVs also required adaptations to overcome restriction by APOBEC3G [22]. In a macaque-specific variant of APOBEC3G, an otherwise conserved Y at position 59 that is present in other Old World primates (APOBEC3G^Y^) was replaced by an LR (APOBEC3G^LR^) [22]. SIVsmm Vifs cannot counteract this variant. An orthologous APOBEC3G^LR^ allele is also found in crab-eating macaques while pig-tailed macaques appear to be homozygous for the ancestral APOBEC3G^Y^ ortholog [22, 47]. To replicate in rhesus macaques, a single amino acid substitution emerged in Vif, G17E, which restores binding to APOBEC3G^LR^ [22]. While Vif G17 is found in nearly all SIVsmm isolates and in SIVstm, Vif E17 is found in SIVmac, SIVmne and in our SIVmfa isolate (Fig 2B). Notably, pig-tailed macaques, the host of SIVmne, don’t appear to encode the APOBEC3G^LR^ allele [22, 47].

Thus, there are three adaptations that seem to be unique to SIV from macaques: CA LPA89-91QQ, CA R97S and Vif G17E. We note that in all instances of cross-species transmission of SIVsmm, including the SIV isolates that arose following the unintentional or the experimental infection of macaques with SIVsmm from both captive and wild sooty mangabeys, and HIV-2 isolates that arose following the transmission of SIVsmm to humans, the combination of these three adaptations is only found in SIVs belonging to the SIVmac/SIVmne/SIVmfa lineage (Fig 2). Importantly, the complex LPA-to-QQ mutation in CA has not been observed outside the SIVmac/SIVmne/SIVmfa lineage despite the extensive passage of select SIVsmm isolates in macaques (Fig 2A) [46], and it may therefore be a particularly strong marker for emergence of a single SIV lineage in rhesus macaques, which was followed by subsequent transmissions to crab-eating macaques and to pig-tailed macaques.

Based on these observations, we propose that rhesus macaques were initially infected with SIVsmm and that this virus then adapted to the rhesus macaque host, which included adaptions to overcome restriction factor variants that are uniquely found at high allelic frequencies in this species, and emerged as SIVmac. While SIVmac, SIVmne and SIVmfa share identical adaptations, the phylogenetic separation between SIVmne and SIVmfa strongly suggests that genetically distinct SIVmac variants were then independently transmitted to crab-eating and pig-tailed macaques on separate occasions (Fig 1). Thus, at the time of transmission to crab-eating and pig-tailed macaques, distinct SIVmac lineages had already been established in captive rhesus macaques in the United States.

### Proposed origins of SIV infection in crab-eating macaques

SIVmac likely originated at CNPRC in the mid-1960s, following the accidental infection of rhesus macaques with SIVsmm [11, 12, 14]. The prevailing hypothesis is that Kuru experiments exposed rhesus macaques to SIV-infected material from sooty mangabeys [13]. SIVmac was then introduced into the rhesus macaque colony at NEPRC in 1970 via a shipment of SIV-infected monkeys from CNPRC [14]. In an attempt to trace the origins of SIVmfa, we surveyed available literature and veterinary records.

In 1986, four crab-eating macaques at NEPRC were found to be sero-reactive to SIVmac antigens (Table 1) [20]. SIVmfa186 was isolated from the single surviving monkey, Mf186-76, which was euthanized a few months later (death: 11/06/1986). The three other monkeys with SIVmac-reactive sera were Mf021-77 (death: 12/25/1980), Mf114-73 (death: 01/06/1976), and Mf222-73 (death: 08/02/1975). The presence of SIVmac-reactive antibodies in Mf222-73 indicates that by 1975 at least one crab-eating macaque was infected with a virus that was antigenically similar to SIVmac. While Mf186-76 and Mf021-77 were born at NEPRC, Mf114-73 and Mf222-73 were acquired from LEMSIP in February and March 1973, respectively. No evidence of SIV infection in the NEPRC crab-eating macaque colony was uncovered prior to these shipments.

**Table 1:** Crab-eating macaques involved in the importation or spread of SIV at NEPRC.

Animal	Born at	DOB	Arrival at NEPRC	DOD	Recipient of blood from	Sero-positivity [20]	sAIDS
Mf107-73	LEMSIP	unknown	02/22/73	01/30/80			
Mf108-73	LEMSIP	unknown	02/22/73	07/08/73			
Mf109-73	LEMSIP	unknown	02/22/73	10/09/74	Mm		+
Mf110-73	LEMSIP	unknown	02/22/73	01/15/74			
Mf111-73	LEMSIP	unknown	02/22/73	12/29/78	Mm		
Mf112-73	LEMSIP	unknown	02/22/73	09/07/73			+
Mf113-73	LEMSIP	unknown	02/22/73	01/29/76	Mf		
Mf114-73	LEMSIP	05/30/72	02/22/73	01/06/76		+	+
Mf115-73	LEMSIP	unknown	02/22/73	12/01/76			
Mf116-73	LEMSIP	08/14/72	02/22/73	10/26/73			+
Mf117-73	LEMSIP	unknown	02/22/73	08/29/76			
Mf118-73	LEMSIP	unknown	02/22/73	12/24/73			
Mf119-73	LEMSIP	unknown	02/22/73	11/08/76			
Mf120-73	LEMSIP	unknown	02/22/73	10/16/78			
Mf121-73	LEMSIP	03/05/70	02/22/73	01/22/79			
Mf122-73	LEMSIP	09/23/71	02/22/73	10/22/74			
Mf221-73	LEMSIP	03/05/70	03/23/73	Shipped			
Mf222-73	LEMSIP	09/23/71	03/23/73	08/02/75		+	+
Mf223-73	LEMSIP	03/26/70	03/23/73	11/12/73			+
Mf224-73	LEMSIP	02/28/71	03/23/73	07/03/74	Mf		+
Mf225-73	LEMSIP	unknown	03/23/73	12/14/75			+
Mf226-73	LEMSIP	08/27/70	03/23/73	04/24/74			+
Mf227-73	LEMSIP	04/30/71	03/23/73	01/30/74			
Mf186-76	NEPRC	05/21/76	n/a	11/06/86		+	+
Mf021-77	NEPRC	02/16/77	n/a	12/25/80		+	+

DOB = date of birth; DOD = date of death; Mf = Macaca fascicularis; Mm = Macaca mulatta; sAIDS = signs consistent with simian AIDS, documented in veterinary records; n/a = not applicable.

At least 23 crab-eating macaques were shipped from LEMSIP to NEPRC between February and March 1973. Records indicate a pattern of high mortality and signs consistent with simian AIDS among these animals (Table 1). By the end of 1973, five of the 23 monkeys were deceased. This includes one animal, Mf116-73, that became ill while quarantined prior to its introduction into the NEPRC colony, remained isolated, and died on 10/26/1973 of multifocal interstitial pneumonia, a manifestation of simian AIDS observed in SIVmac-infected rhesus macaques [48–50]. Within a year of their arrival at NEPRC, seven of 23 animals had died and by two years, eleven of 23 had died. Of these eleven, seven died directly of or with disease manifestations consistent with simian AIDS. In total, nine of the 23 crab-eating macaques displayed pathologies similar to those observed in SIVmac-infected rhesus macaques.

We propose that the likely origins of SIVmfa were at LEMSIP. One motivation for the establishment of LEMSIP by Dr. Jan Moor-Jankowski and Edward Goldsmith was to provide a sufficiently large and diverse primate colony to support ongoing efforts to define and trace the evolution of the blood antigens of apes, monkeys and humans [51–53]. These studies relied on reactive sera, which were generated by inoculating animals with the blood of other members of the same or different species (often adjuvanted) [51, 52, 54–57]. Published reports indicate that LEMSIP housed both rhesus macaques and crab-eating macaques and furthermore that some crab-eating macaques were inoculated with rhesus macaque blood [55, 56]. The accompanying veterinary records for the 23 animals shipped to NEPRC indicate that at least two of these animals (Mf109-73 and Mf111-73) were inoculated with rhesus macaque blood at LEMSIP (Table 1). Mf109-73 died from lymphoma, which is characteristic of late-stage SIV infection, roughly 2.5 years after such an inoculation [16, 48, 49, 58]. LEMSIP veterinary records also documented transfusions of blood between crab-eating macaques (Table 1). In contrast, we did not uncover any evidence of crab-eating macaques being exposed to or housed with rhesus macaques at NEPRC. However, we uncovered a number of documented blood transfusions between apparently healthy and anemic/wasting crab-eating macaques. We determined that Mf109-73, which we suspect was SIV-positive, was a blood donor to another crab-eating macaque (Mf292-72). Mf109-73 was also housed with other crab-eating macaques as part of the existing NEPRC colony and was mated with a sire (Mf339-67). Veterinary records for these two additional animals were not located.

Importantly, these events occurred prior to the discovery of primate lentiviruses, and workers at LEMSIP and the other National Primate Research Centers would have been unaware of the existence of SIV in their colonies. These findings suggest the emergence of SIVmfa began with unintentional infection of crab-eating macaques at LEMSIP.

## Discussion

Early events in the emergence of HIV-1 occurred in Africa during the early to mid-1900s, and archived samples from this period of adaptation are either rare or non-existent. The establishment of primate centers in the 20^th^ century provided the opportunity for transmission of SIV from African sooty mangabeys to Asian macaques, and experimentation and husbandry practices likely spread ill-adapted SIVsmm virus among macaques until pathogenic SIVs emerged [13]. Because these SIVs emerged in captive animals, undiscovered archived samples and veterinary records from this period of viral adaption may still exist. To this end, we sought to recover the sequence of a “lost” SIV from crab-eating macaques and infer its origins.

We believe that SIVmfa originated at LEMSIP in the early 1970s and was then spread to NEPRC in 1973. These conclusions are based on compelling evidence: 1) two of the four known SIV-positive crab-eating macaques were shipped from LEMSIP to NEPRC in 1973; 2) these are the two oldest confirmed cases of SIV in crab-eating macaques; 3) other monkeys in these shipments died with signs consistent with sAIDS; 4) crab-eating macaques were infused with rhesus macaque blood at LEMSIP; 5) by 1970, SIVmac was already present in at least two primate centers, CNPRC and NEPRC [12, 14]; 6) no documented case of exposure of crab-eating macaques to rhesus macaque blood was uncovered at NEPRC. Thus, like the Kuru experiments carried out at CNPRC that likely resulted in the emergence of SIVmac [13], investigations into primate blood antigens carried out at LEMSIP most likely resulted in the emergence of SIVmfa.

Following its introduction into the NEPRC crab-eating macaque colony, SIVmfa likely persisted in a small number of animals until its eradication in 1986. A total of four confirmed SIV-positive crab-eating macaques had died in 1975, 1976, 1980 and 1986, respectively [20]. This number is not dissimilar to the dozen or so known SIVmac-infected rhesus macaques from 1970 to 1986 [14, 16, 20, 59]. Including SIV-infected animals unknowingly acquired by NEPRC, less than 20 macaques of any species are known to have been unintentionally infected with SIV between 1970 and 1986 at NEPRC [14, 16, 20]. It is therefore unlikely that there were multiple independent cross-species transmissions of SIVmac to crab-eating macaques at NEPRC. Furthermore, it suggests that veterinary and husbandry practices at NEPRC did not readily spread SIV.

**Fig 3:**
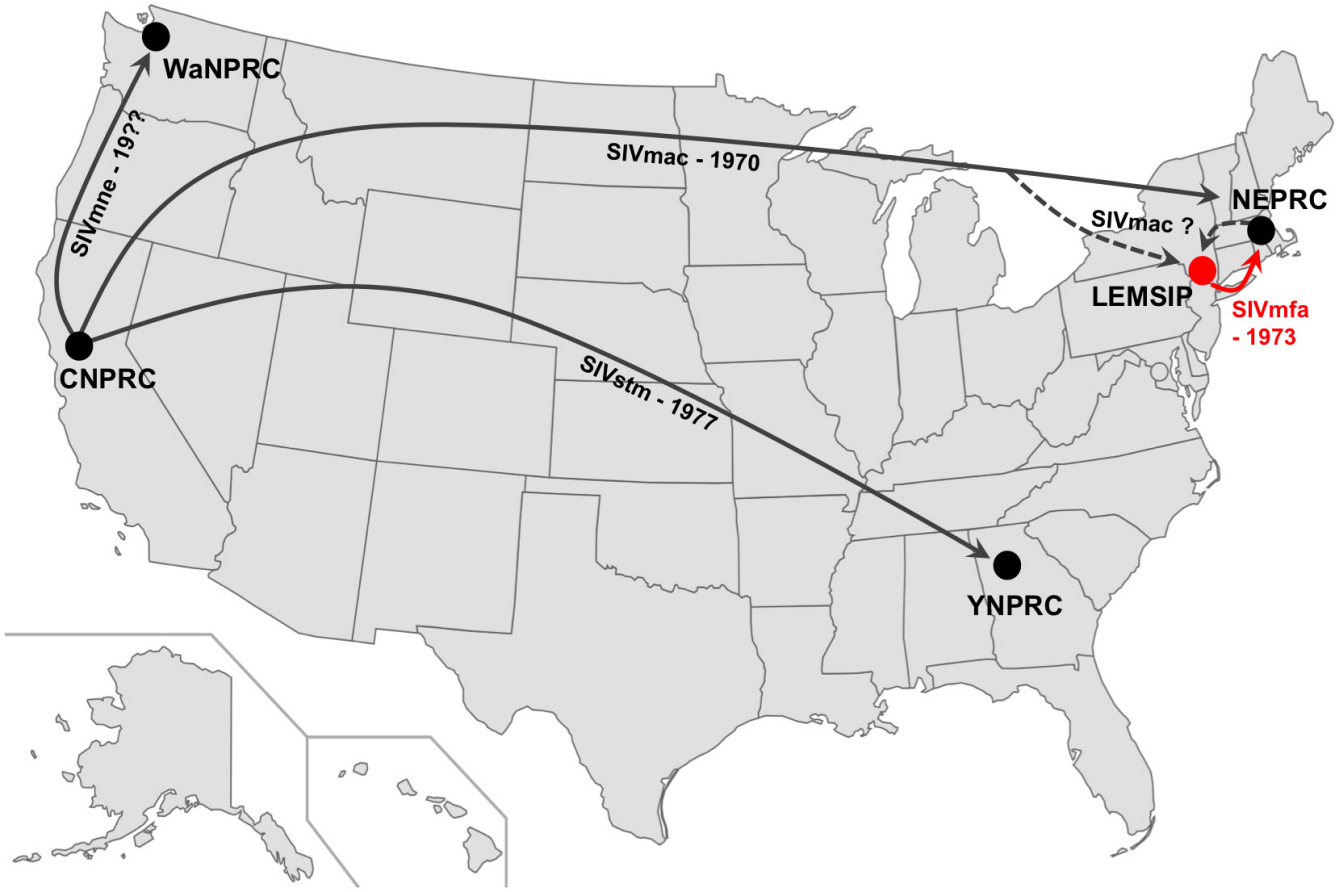
Proposed origin of SIVmfa in United States primate facilities. Shipments of SIV-infected macaques in the continental United States with approximate times are indicated by arrows. LEMSIP and the shipment of SIVmfa-infected animals are highlighted in red. Dashed lines are speculative and indicate possible sources of SIVmac at LEMSIP. WaNPRC = Washington National Primate Research Center.

Our analysis has allowed us to resolve the series of cross-species transmission events that gave rise to SIVmac, SIVmne and SIVmfa. Based on our phylogenetic analyses and the identification of adaptations that specifically overcome host restriction factor variants present at high frequencies only in rhesus macaques (including a unique deletion-mutation at capsid positions 89–91; Fig 2), we conclude that rhesus macaques were initially infected with SIVsmm, which underwent adaptation to rhesus macaques followed by transmission to pig-tailed and crab-eating macaques on separate occasions. This conclusion is consistent with the known history of macaque SIVs (Fig 3) [12–14, 58]. SIVsmm was unintentionally transmitted to rhesus macaques at CNPRC between 1964 (the arrival of sooty mangabeys at CNPRC) and no later than 1970, when infected animals were shipped to NEPRC [12–14, 58]. The dramatic increase in sAIDS-associated opportunistic infections at CNPRC that likely signified the emergence of pathogenic SIVmac began in 1969, within five years of the earliest possible cross-species transmission event. Despite its circulation in rhesus macaque colonies, SIVmac was not identified until 1985 by researches at NEPRC [20]. While hypothesized to have been present at the Washington National Primate Research Center (WaNPRC) by 1976 [17], the earliest isolate of SIVmne is from 1982 [17]. We believe crab-eating macaques were infected with SIVmac in the early 1970s at LEMSIP, with the first confirmed case of SIVmfa identified at NEPRC in 1975 (Fig 3) [20].

We propose that following the transmission of SIVmac to crab-eating macaques, SIVmfa had a period of 10+ years to adapt its new host. Adaptation was likely required since experimental infections of crab-eating macaques with SIVmac239/SIVmac251 often result in lower viral loads, higher instances of spontaneous control and slower disease onset than is observed in rhesus macaques [60, 61]. The sequence of SIVmfa may therefore aid efforts to improve models of SIV infection in crab-eating macaques, including those in Mauritian-origin crab-eating macaques. These macaques are well suited for large vaccine studies because unlike other macaques they have extremely limited MHC diversity [62, 63]. Compared to SIVmac or SIVmne, SIVmfa may have adaptations within known crab-eating macaque CTL epitopes [64–66], and therefore SIVmfa may replicate more uniformly in these animals.

## Conclusions

Isolation of SIVmfa from crab-eating macaques demonstrates that SIV infection of captive macaques was more widespread than is generally appreciated. Our findings raise the distinct possibility that other SIV isolates may have been present at other institutions that housed macaques from the mid 1960s through the late 1980s. Such samples could prove to be invaluable for documenting the history of SIV infection and studying the process of cross-species transmission, viral adaptation, and emergence of novel pathogens. Moreover, vaccine approaches and challenge stocks based on SIVmfa may aid the development of the Mauritian-origin crab-eating macaque model system.

## Supporting Information

S1 Fig Single-gene phylogenies of the structural and enzymatic genes of SIVmfa and related SIV and HIV-2 sequences. (TIF)

S2 Fig Single-gene phylogenies of the accessory genes of SIVmfa and related SIV and HIV-2 sequences. (TIF)

S3 Fig Phylogenies of partial gene sequences of SIVmfa and related SIV and HIV-2 sequences. (TIF)

S1 Table RT-PCR primers used to amplify SIVmfa from viral RNA isolated from a co-culture of SIVmfa-positive lymphocytes and H9 cells. (TIF)

S2 Table Genbank accession numbers and isolate names of viral sequences used for the genetic and phylogenetic analyses of SIVmfa. (TIF)
