# Supplementary figures and images for: Phylogeny and History of the Lost SIV from Crab-Eating Macaques: SIVmfa
Source: PLoS One. 2016 Jul 14;11(7):e0159281. doi: 10.1371/journal.pone.0159281 (PMC4944941; doi:10.1371/journal.pone.0159281)

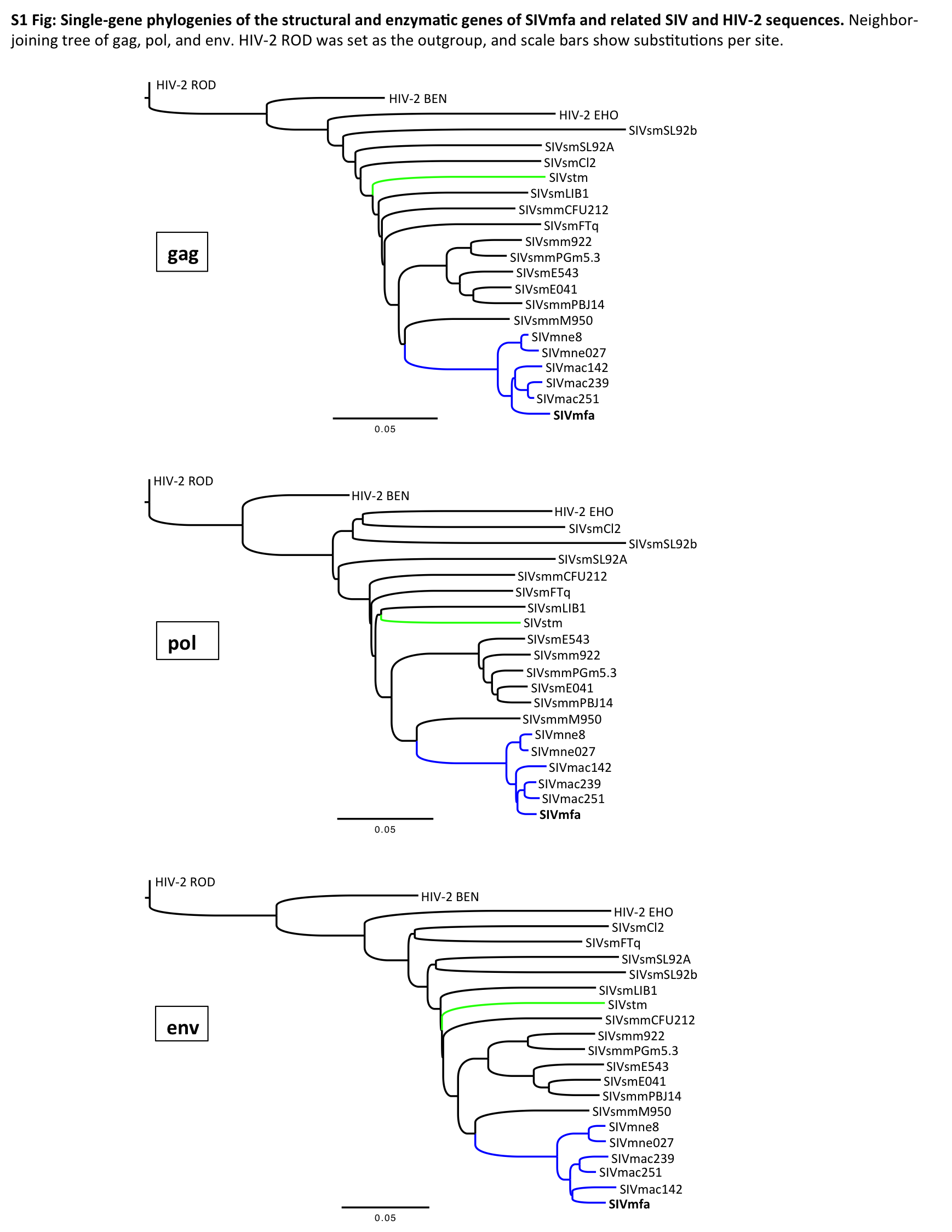

Supplement: S1 Fig — (TIF) [file pone.0159281.s001.tif]

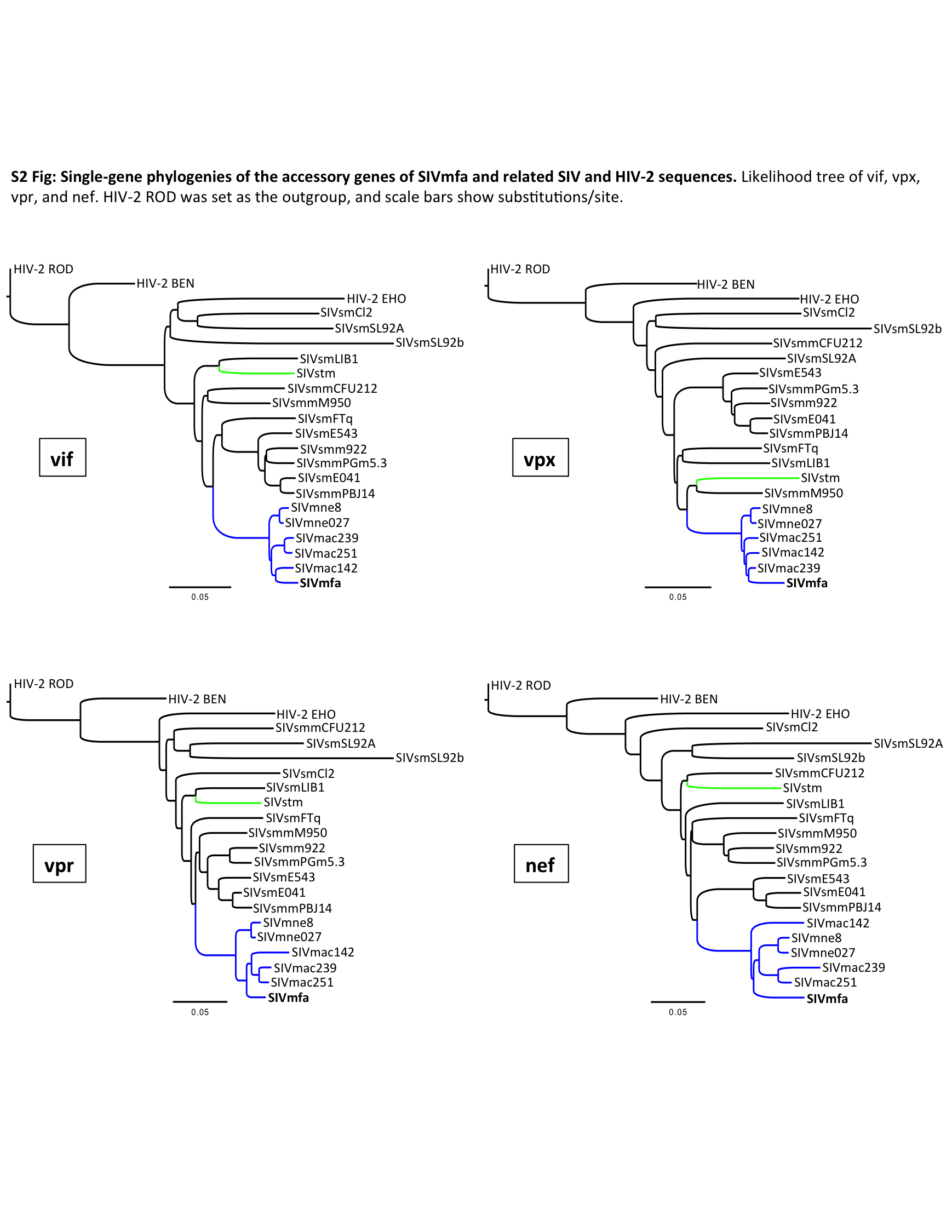

Supplement: S2 Fig — (TIF) [file pone.0159281.s002.tif]

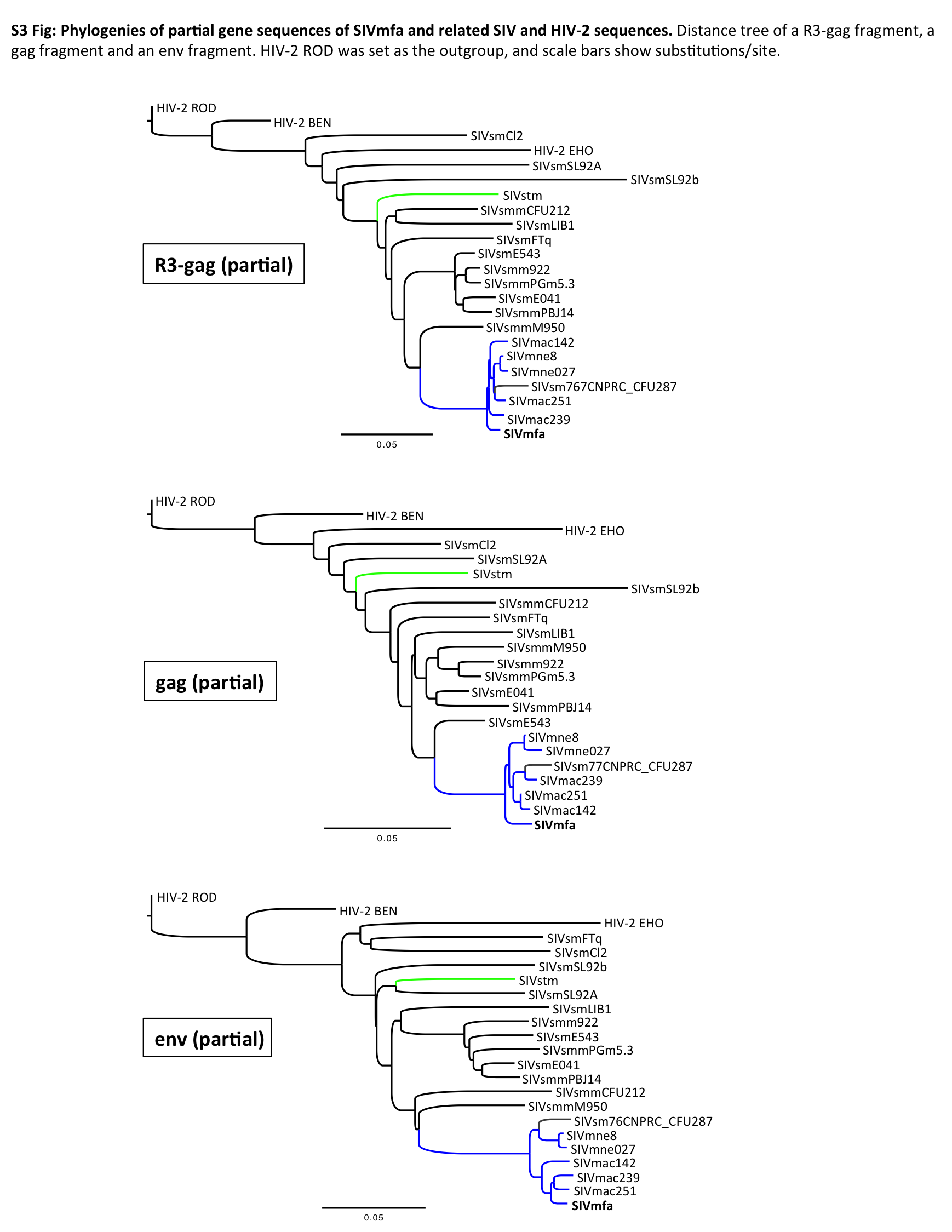

Supplement: S3 Fig — (TIF) [file pone.0159281.s003.tif]

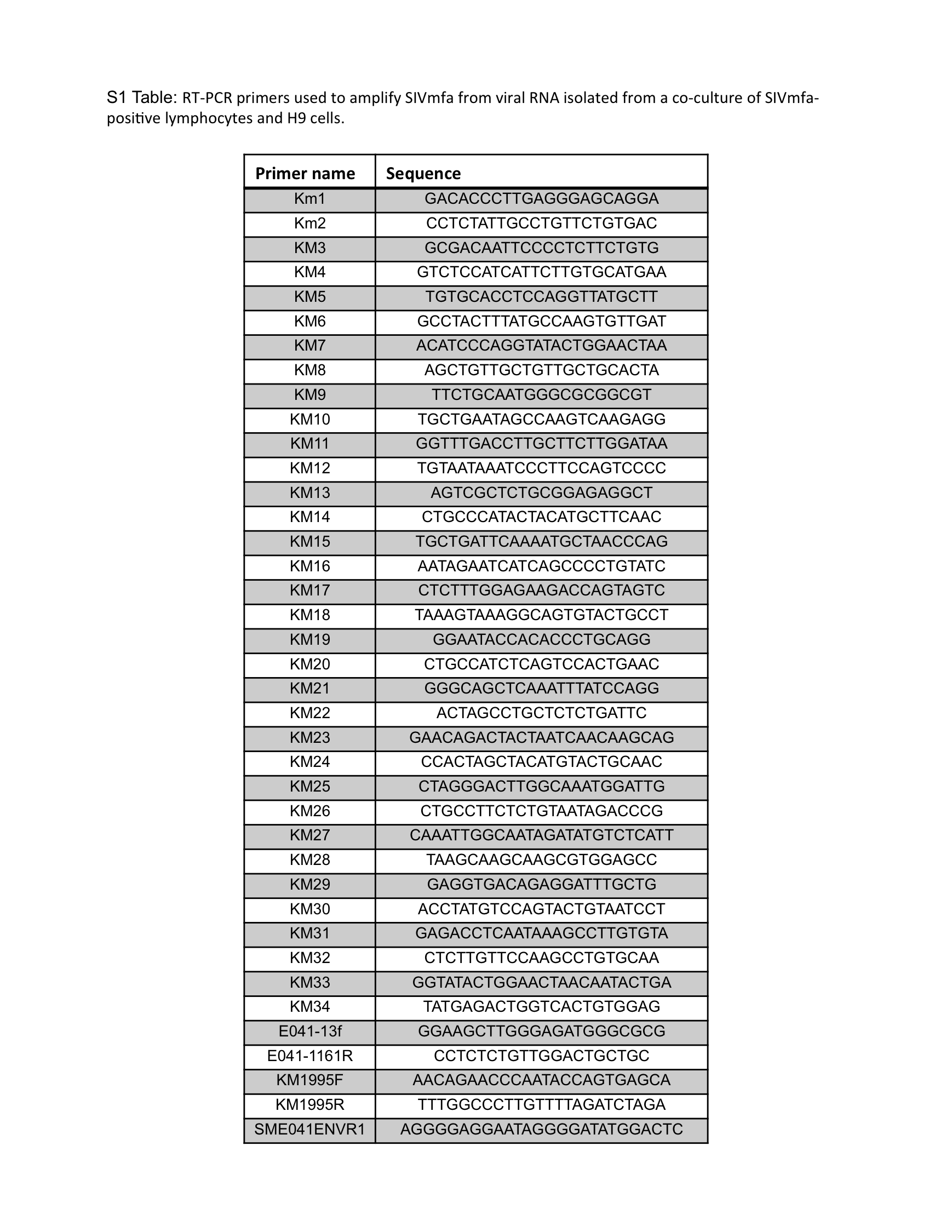

Supplement: S1 Table — (TIF) [file pone.0159281.s004.tif]

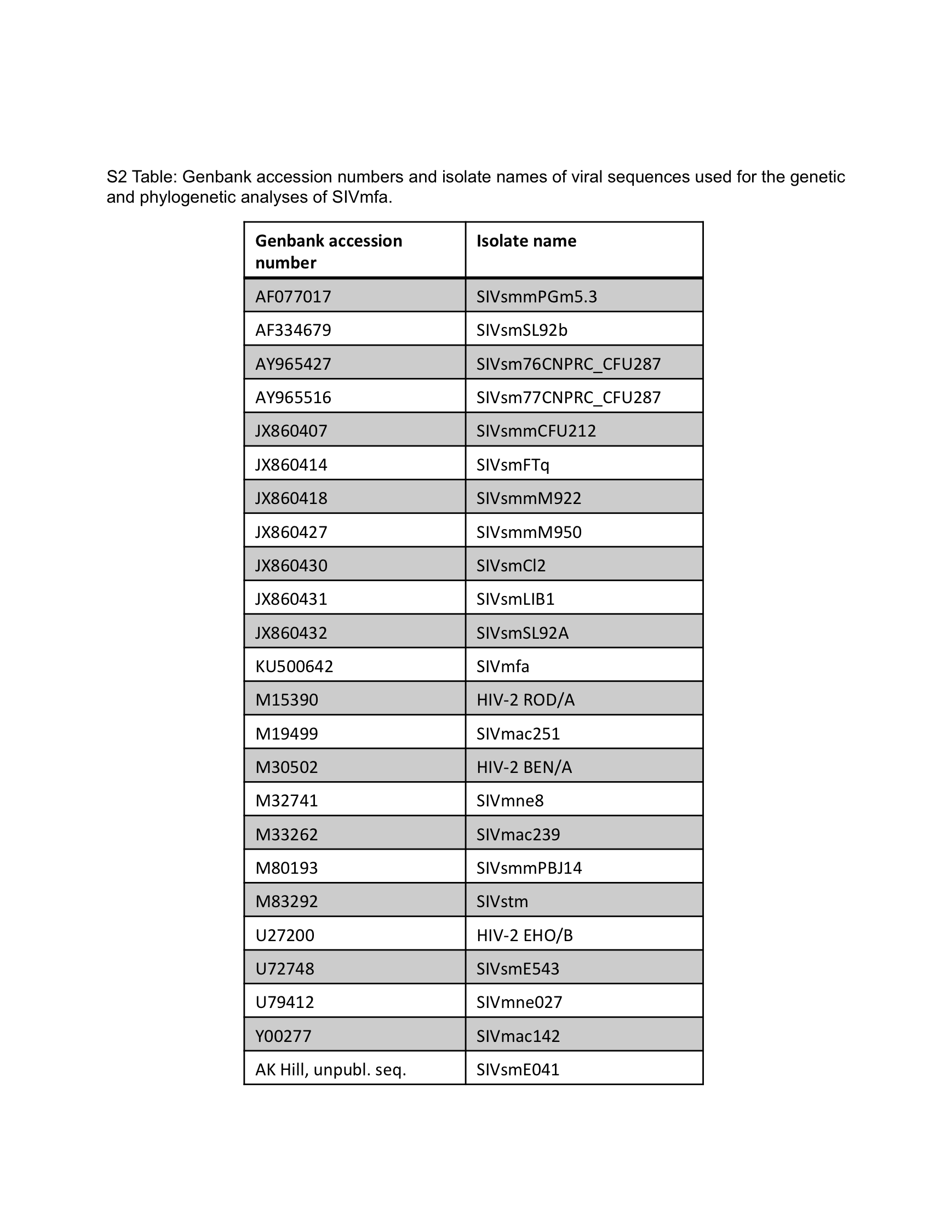

Supplement: S2 Table — (TIF) [file pone.0159281.s005.tif]
